# PreAcrs: a machine learning framework for identifying anti-CRISPR proteins

**DOI:** 10.1186/s12859-022-04986-3

**Published:** 2022-10-25

**Authors:** Lin Zhu, Xiaoyu Wang, Fuyi Li, Jiangning Song

**Affiliations:** 1 Institute for Advanced Study, Shenzhen University, Shenzhen, China; 2 Monash Biomedicine Discovery Institute and Department of Biochemistry and Molecular Biology, Monash University, Melbourne, VIC 3800 Australia; 3 Department of Microbiology and Immunology, The Peter Doherty Institute for Infection and Immunity, The University of Melbourne, Melbourne, VIC Australia; 4 Monash Data Futures Institute, Monash University, Melbourne, VIC 3800 Australia

**Keywords:** Anti-CRISPR protein, Machine learning, Feature engineering, Sequence analysis

## Abstract

**Background:**

Anti-CRISPR proteins are potent modulators that inhibit the CRISPR-Cas immunity system and have huge potential in gene editing and gene therapy as a genome-editing tool. Extensive studies have shown that anti-CRISPR proteins are essential for modifying endogenous genes, promoting the RNA-guided binding and cleavage of DNA or RNA substrates. In recent years, identifying and characterizing anti-CRISPR proteins has become a hot and significant research topic in bioinformatics. However, as most anti-CRISPR proteins fall short in sharing similarities to those currently known, traditional screening methods are time-consuming and inefficient. Machine learning methods could fill this gap with powerful predictive capability and provide a new perspective for anti-CRISPR protein identification.

**Results:**

Here, we present a novel machine learning ensemble predictor, called PreAcrs, to identify anti-CRISPR proteins from protein sequences directly. Three features and eight different machine learning algorithms were used to train PreAcrs. PreAcrs outperformed other existing methods and significantly improved the prediction accuracy for identifying anti-CRISPR proteins.

**Conclusions:**

In summary, the PreAcrs predictor achieved a competitive performance for predicting new anti-CRISPR proteins in terms of accuracy and robustness. We anticipate PreAcrs will be a valuable tool for researchers to speed up the research process. The source code is available at: https://github.com/Lyn-666/anti_CRISPR.git.

**Supplementary Information:**

The online version contains supplementary material available at 10.1186/s12859-022-04986-3.

## Background

CRISPR-Cas adaptive immune system is one of the most widespread immunity strategies in prokaryotes against invading bacteriophages and plasmids [1, 2]. To counteract and overcome different CRISPR-Cas immunity systems, bacteriophages have evolved anti-CRISPR proteins (Acrs) that were first discovered in *Pseudomonas aeruginosa* phages in 2013 [3]. Subsequently, a proliferation of Acrs has proved to inactivate multiple CRISPR subtypes [3–7].

Several methods have been proposed to identify Acrs, including “Guilt-by-association” studies [6, 8], self-targeting CRISPR arrays [6, 7], and metagenome DNA screening [9, 10], etc. These methods assumed the new Acrs are similar to the previous Acrs. However, most Acrs fall short in sharing similarities currently acknowledged. Therefore, the traditional screening methods based on homology search are unreliable and require a lot of prior knowledge of Acrs to identify new Acrs. For instance, the “Guilt-by-association” method involves searching for homologs of helix-turn-helix (HTH)-containing proteins that are typically encoded downstream of Acrs [11]. The performance of “Guilt-by-association” is unstable when known Acrs proteins might share low similarity with queried protein. Therefore, a computational approach with less requirement for prior knowledge of known Acrs will provide a new perspective on the identification of Acrs. Machine learning algorithms with appropriate features could reveal the potential mechanism of Acrs and identify the Acrs without prior knowledge.

Recently, some machine learning methods have been presented for predicting Acrs. There are several web servers about Acrs, such as: Anti-CRISPRdb [12], AcrHub [13], AcrDB [14], CRISPRminer2 [15], AcRanker [14, 16], AcrFinder [17], AcrCatalog [18] and PaCRISPR [19]. Anti-CRISPRdb, AcrDB, and AcrCatalog are online Acr datasets, while AcrHub, CRISPRminer2, AcRanker, AcrFinder and PaCRISPR are prediction web servers. Eitzinger et al. developed AcRanker, using the XGBoost ranking model to predict candidate Acrs only based on protein sequence information [16]. Wang et al. proposed PaCRISPR, an ensemble learning-based predictor, to identify Acrs from protein datasets derived from genome and metagenome sequencing projects [19]. Gussow et al. proposed a machine learning approach, using a random forest model with extremely randomized trees to expand the repertoire of Acrs families [20]. These machine learning methods have made a great contribution to discovering Acrs. However, the most appropriate features or feature combinations for Acrs prediction have not been systematically assessed. For instance, The PaCRISPR method identified the Acrs using only evolutionary features, and the AcRanker used only amino acid composition features to identify Acrs. Gussow et al. predict Acrs based on the sequence alignment and a heuristic secondary screen of few known Acrs. Thus, since previous work did not fully assess the feature combinations and relied on prior knowledge, we proposed a novel, effective and robust machine learning framework to help identify Acrs.

This study presented an ensemble machine learning method, called PreAcrs, to efficiently and accurately predict Acrs based on protein sequences. Specifically, we used three features and eight different machine learning methods to train our model. 412 experimentally validated Acrs and 412 non-Acrs were introduced in the training dataset, and 176 were experimentally determined Acrs and 176 non-Acrs in the independent dataset. We found that the PreAcrs method outperformed other existing predictors with an AUC of 0.972 in the independent dataset.

## Results and discussion

### Performance evaluation of five different features

To find the appropriate feature encoding methods, we evaluated and compared the performance of nine machine learning methods, including SVM, KNN, MLP, LR, RF, XGBoost, LightGBM, CatBoost and ensemble methods, for each feature encoding based on a randomized fivefold cross-validation. The results of classifiers based on the fivefold cross-validation are shown in Table 1.

**Table 1:** Performance comparison of different features and classifiers based on the fivefold cross-validation

Feature	Model	PRE	SN	SP	F-score	ACC	MCC
AAC	SVM	**0.874 ± 0.077**	0.638 ± 0.175	**0.886 ± 0.116**	0.716 ± 0.096	0.762 ± 0.050	**0.562 ± 0.080**
	KNN	0.762 ± 0.035	**0.808 ± 0.063**	0.742 ± 0.071	**0.782 ± 0.017**	**0.775 ± 0.015**	0.557 ± 0.032
	RF	0.669 ± 0.041	0.621 ± 0.091	0.689 ± 0.082	0.640 ± 0.054	0.655 ± 0.040	0.314 ± 0.078
	MLP	0.790 ± 0.071	0.704 ± 0.128	0.794 ± 0.116	0.732 ± 0.054	0.749 ± 0.033	0.514 ± 0.059
	LR	0.743 ± 0.051	0.738 ± 0.124	0.733 ± 0.118	0.732 ± 0.055	0.735 ± 0.040	0.483 ± 0.075
	XGB	0.793 ± 0.071	0.718 ± 0.077	0.801 ± 0.094	0.749 ± 0.034	0.760 ± 0.033	0.528 ± 0.072
	Light	0.800 ± 0.069	0.670 ± 0.076	0.820 ± 0.086	0.723 ± 0.023	0.745 ± 0.016	0.503 ± 0.043
	CAT	0.785 ± 0.052	0.745 ± 0.063	0.791 ± 0.069	0.762 ± 0.039	0.768 ± 0.038	0.540 ± 0.077
	Ens_vote	0.826 ± 0.058	0.745 ± 0.085	0.835 ± 0.083	0.779 ± 0.043	0.790 ± 0.036	0.589 ± 0.072
	Sta_LR	0.837 ± 0.048	0.745 ± 0.080	0.849 ± 0.066	0.785 ± 0.038	0.797 ± 0.030	0.603 ± 0.060
	Sta_GBC	0.818 ± 0.064	0.701 ± 0.096	0.837 ± 0.078	0.750 ± 0.058	0.769 ± 0.046	0.550 ± 0.092
PAAC	SVM	0.869 ± 0.054	0.658 ± 0.140	0.900 ± 0.047	0.741 ± 0.103	0.779 ± 0.069	0.580 ± 0.124
	KNN	0.711 ± 0.030	**0.934 ± 0.031**	0.616 ± 0.063	0.807 ± 0.017	0.775 ± 0.025	0.583 ± 0.046
	RF	0.808 ± 0.058	0.758 ± 0.122	0.811 ± 0.093	0.774 ± 0.068	0.784 ± 0.050	0.578 ± 0.090
	MLP	**0.893 ± 0.067**	0.614 ± 0.143	**0.917 ± 0.061**	0.714 ± 0.098	0.766 ± 0.058	0.566 ± 0.094
	LR	0.748 ± 0.032	0.760 ± 0.153	0.735 ± 0.088	0.743 ± 0.069	0.748 ± 0.040	0.509 ± 0.080
	XGB	0.841 ± 0.048	0.748 ± 0.120	0.854 ± 0.053	0.785 ± 0.069	0.801 ± 0.049	0.612 ± 0.091
	Light	0.848 ± 0.048	0.760 ± 0.105	0.859 ± 0.055	0.796 ± 0.059	0.810 ± 0.044	0.628 ± 0.083
	CAT	0.856 ± 0.047	0.801 ± 0.112	0.861 ± 0.055	**0.823 ± 0.064**	**0.831 ± 0.051**	**0.670 ± 0.097**
	Ens_vote	0.871 ± 0.043	0.770 ± 0.122	0.881 ± 0.053	0.810 ± 0.065	0.825 ± 0.046	0.662 ± 0.082
	Sta_LR	0.866 ± 0.046	0.745 ± 0.131	0.881 ± 0.051	0.794 ± 0.076	0.813 ± 0.056	0.640 ± 0.102
	Sta_GBC	0.875 ± 0.035	0.719 ± 0.081	0.896 ± 0.035	0.786 ± 0.050	0.807 ± 0.037	0.627 ± 0.068
PSSM-AC	SVM	0.776 ± 0.389	0.226 ± 0.186	0.990 ± 0.014	0.327 ± 0.240	0.608 ± 0.087	0.298 ± 0.190
	KNN	0.821 ± 0.042	**0.828 ± 0.046**	0.818 ± 0.048	**0.824 ± 0.033**	**0.823 ± 0.033**	**0.647 ± 0.068**
	RF	0.881 ± 0.058	0.359 ± 0.092	0.954 ± 0.019	0.505 ± 0.102	0.657 ± 0.049	0.387 ± 0.094
	MLP	**1.000 ± 0.000**	0.231 ± 0.087	**1.000 ± 0.000**	0.367 ± 0.110	0.615 ± 0.045	0.357 ± 0.076
	LR	0.952 ± 0.035	0.507 ± 0.184	0.971 ± 0.025	0.640 ± 0.157	0.739 ± 0.083	0.543 ± 0.130
	XGB	0.936 ± 0.042	0.352 ± 0.108	0.976 ± 0.020	0.502 ± 0.117	0.664 ± 0.051	0.418 ± 0.088
	Light	1.000 ± 0.000	0.272 ± 0.064	**1.000 ± 0.000**	0.424 ± 0.083	0.636 ± 0.033	0.395 ± 0.057
	CAT	0.957 ± 0.032	0.424 ± 0.140	0.976 ± 0.026	0.572 ± 0.121	0.700 ± 0.057	0.483 ± 0.080
	Ens_vote	0.988 ± 0.014	0.328 ± 0.090	0.995 ± 0.006	0.485 ± 0.106	0.661 ± 0.043	0.432 ± 0.069
	Sta_LR	0.981 ± 0.016	0.338 ± 0.081	0.993 ± 0.006	0.496 ± 0.097	0.665 ± 0.040	0.436 ± 0.064
	Sta_GBC	0.977 ± 0.023	0.347 ± 0.141	0.990 ± 0.009	0.496 ± 0.147	0.669 ± 0.068	0.438 ± 0.107
RPSSM	SVM	0.914 ± 0.062	0.713 ± 0.229	0.915 ± 0.068	0.767 ± 0.172	0.814 ± 0.090	0.659 ± 0.133
	KNN	0.738 ± 0.020	**0.925 ± 0.024**	0.670 ± 0.035	0.820 ± 0.014	0.797 ± 0.016	0.616 ± 0.031
	RF	0.922 ± 0.015	0.694 ± 0.102	0.939 ± 0.020	0.787 ± 0.063	0.817 ± 0.042	0.657 ± 0.064
	MLP	0.870 ± 0.034	0.898 ± 0.016	0.864 ± 0.040	**0.883 ± 0.017**	**0.881 ± 0.019**	**0.763 ± 0.038**
	LR	0.815 ± 0.066	0.876 ± 0.069	0.789 ± 0.105	0.840 ± 0.027	0.833 ± 0.033	0.676 ± 0.057
	XGB	0.892 ± 0.017	0.777 ± 0.078	0.905 ± 0.021	0.828 ± 0.045	0.841 ± 0.034	0.690 ± 0.058
	Light	0.907 ± 0.013	0.767 ± 0.092	0.920 ± 0.020	0.828 ± 0.053	0.843 ± 0.039	0.698 ± 0.066
	CAT	**0.926 ± 0.014**	0.765 ± 0.066	**0.939 ± 0.011**	0.836 ± 0.042	0.852 ± 0.034	0.716 ± 0.061
	Ens_vote	0.913 ± 0.026	0.849 ± 0.047	0.917 ± 0.032	0.879 ± 0.021	0.883 ± 0.017	0.771 ± 0.032
	Sta_LR	0.921 ± 0.020	0.844 ± 0.052	0.927 ± 0.019	0.880 ± 0.031	0.886 ± 0.026	0.775 ± 0.049
	Sta_GBC	0.892 ± 0.035	0.820 ± 0.025	0.898 ± 0.039	0.854 ± 0.010	0.859 ± 0.012	0.722 ± 0.026
SSA	SVM	0.903 ± 0.038	0.740 ± 0.099	0.915 ± 0.043	0.807 ± 0.054	0.828 ± 0.033	0.671 ± 0.047
	KNN	0.699 ± 0.036	**0.937 ± 0.043**	0.592 ± 0.070	0.799 ± 0.028	0.765 ± 0.037	0.566 ± 0.071
	RF	0.840 ± 0.016	0.663 ± 0.068	0.874 ± 0.018	0.739 ± 0.043	0.768 ± 0.029	0.550 ± 0.052
	MLP	0.881 ± 0.046	0.772 ± 0.146	0.886 ± 0.070	**0.811 ± 0.073**	**0.829 ± 0.046**	**0.675 ± 0.075**
	LR	0.817 ± 0.036	0.811 ± 0.113	0.813 ± 0.062	0.808 ± 0.053	0.812 ± 0.037	0.633 ± 0.072
	XGB	0.858 ± 0.018	0.731 ± 0.112	0.879 ± 0.028	0.784 ± 0.064	0.805 ± 0.046	0.620 ± 0.083
	Light	**0.908 ± 0.038**	0.624 ± 0.123	**0.934 ± 0.034**	0.732 ± 0.083	0.779 ± 0.054	0.591 ± 0.089
	CAT	0.882 ± 0.021	0.745 ± 0.119	0.898 ± 0.033	0.802 ± 0.063	0.822 ± 0.045	0.657 ± 0.080
	Ens_vote	0.887 ± 0.026	0.787 ± 0.119	0.898 ± 0.034	0.828 ± 0.069	0.842 ± 0.051	0.695 ± 0.092
	Sta_LR	0.903 ± 0.033	0.738 ± 0.118	0.917 ± 0.045	0.806 ± 0.069	0.828 ± 0.048	0.672 ± 0.083
	Sta_GBC	0.882 ± 0.080	0.597 ± 0.112	0.915 ± 0.060	0.705 ± 0.083	0.756 ± 0.060	0.545 ± 0.115
RPSSM&PSSM_AC&SSA	SVM	**0.974 ± 0.020**	0.713 ± 0.155	0.978 ± 0.018	0.811 ± 0.115	0.846 ± 0.071	0.722 ± 0.110
	KNN	0.826 ± 0.015	**0.917 ± 0.033**	0.806 ± 0.022	**0.869 ± 0.017**	0.862 ± 0.015	0.729 ± 0.033
	RF	0.966 ± 0.014	0.665 ± 0.093	0.976 ± 0.013	0.784 ± 0.062	0.820 ± 0.042	0.676 ± 0.066
	MLP	0.969 ± 0.021	0.740 ± 0.110	0.973 ± 0.019	0.833 ± 0.067	0.857 ± 0.046	0.738 ± 0.069
	LR	0.927 ± 0.029	0.796 ± 0.078	0.934 ± 0.031	0.853 ± 0.041	**0.865 ± 0.028**	**0.741 ± 0.045**
	XGB	0.961 ± 0.013	0.699 ± 0.084	0.971 ± 0.012	0.806 ± 0.051	0.835 ± 0.037	0.698 ± 0.060
	Light	0.972 ± 0.008	0.595 ± 0.091	**0.983 ± 0.006**	0.734 ± 0.066	0.789 ± 0.044	0.628 ± 0.070
	CAT	0.965 ± 0.009	0.730 ± 0.106	0.973 ± 0.009	0.827 ± 0.070	0.852 ± 0.049	0.728 ± 0.082
	Ens_vote	0.970 ± 0.002	0.774 ± 0.045	0.976 ± 0.000	0.860 ± 0.028	0.875 ± 0.022	0.766 ± 0.039
	Sta_LR	0.978 ± 0.015	0.750 ± 0.047	0.983 ± 0.012	0.848 ± 0.031	0.866 ± 0.025	0.754 ± 0.043
	Sta_GBC	0.982 ± 0.012	0.662 ± 0.058	0.988 ± 0.008	0.790 ± 0.044	0.825 ± 0.030	0.688 ± 0.051

The bold values indicate the best performance

**Fig. 1:**
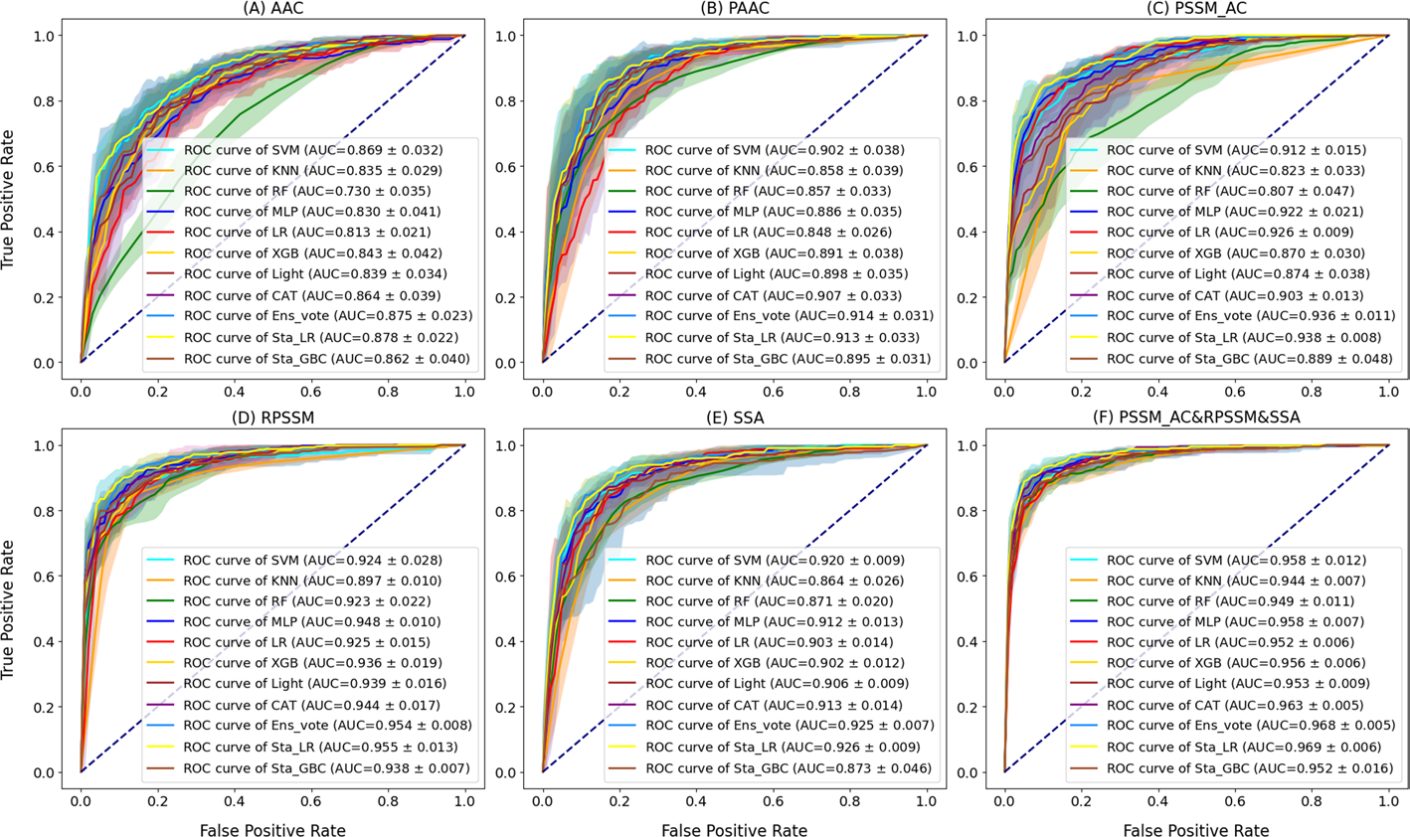
The ROC curve of five single features and AAC&PAAC&RPSSM feature on five-fold cross-validation

We used five feature encoding methods (AAC, PAAC, PSSM_AC, RPSSM, SSA) to convert each protein into a feature vector. As the most forceful one in five feature encoding methods, RPSSM achieved the highest AUC value in eight classifiers (Fig. 1). An interesting phenomenon is that the RPSSM feature obtained the best performance among five single features and the performance of PSSM_AC is second only to RPSSM. The evolutionary features derived from the PSSM files showed that evolutionary features have an outstanding contribution to Acrs prediction. The evolutionary feature RPSSM had a better performance than the evolutionary feature PSSM AC in most classifiers (except LR). The pre-trained machine learning feature SSA also achieved good performance for most classifiers, and its performance is better than sequence features AAC and PAAC. The PAAC contains more sequence information, showing higher AUC values than AAC for all classifiers. The sequence features AAC and PAAC achieved a relatively poor performance compared with other features. One explanation is that evolutionary features and the pre-trained feature encoded more valuable and appropriate information about protein sequences. In contrast, sequence features might involve redundant information that reduces the accuracy of Acrs prediction. In the PreAcrs model, features PAAC_AC, RPSSM and SSA were considered. From Additional file 2: Table S2, the RPSSM-based model achieved the best prediction performance among the three features on the independent test, the PSSM_AC-based model achieved the second prediction accuracy, and the SSA-based model showed a lower prediction accuracy compared to another two features. In addition, the AUC value of the PSSM_AC&SSA was 0.953, up to 0.969 after considering the feature RPSSM. Two ensemble features PSSM_AC&RPSSM and RPSSM&SSA achieved an excellent performance in terms of AUC (0.967 and 0.961, respectively). Therefore, the feature RPSSM made the most contribution to the PreAcrs model in predicting Acrs.

### Performance evaluation of eight different single classifiers and ensemble classifiers

For most feature encodings, the LightGBM classifier, CatBoost and SVM classifier outperformed the other single classifiers (except the ensemble classifier) in terms of PRE (Table 1). This observation is supported by Fernandez-Delgado et al. [21], who found the SVM model is most likely the best classifier compared with the other 17 machine learning methods based on various public data sets. Moreover, Ke et al. [22] demonstrated LightGBM model achieved a better performance than others in multiple public datasets. LightGBM could handle the high-dimension features and large-scale data [22]. CatBoost is proved superior to XGB and LightGBM in terms of a set of publicly available datasets [23]. Although LightGBM obtained the highest PRE values among the eight classifiers in PSSM_AC and SSA in this study, CatBoost had a better performance than LightGBM in RPSSM. In addition, Catboost showed excellent performance in other metrics, such as AUC and MCC. SVM obtained the highest PRE values among the eight classifiers in features AAC and PAAC. It implied that the SVM, LightGBM and CatBoost classifiers provided an outstanding prediction ability, and SVM tended to show excellent performances in sequence features. Additionally, the highest PRE value of 1.00 was obtained by LightGBM classifier when the PSSM_AC feature was used for training during experiments. It means that the predicted positive samples of this model are more likely to be true positive samples, and it might be beneficial for the virtual screening of Acrs.

To fairly compare the performance of various classifiers, other measurements were considered, such as SP, SN, and MCC. As one crucial evaluation matrix, MCC considers all four confusion matrices and can comprehensively reflect the performance. CatBoost presented its powerful and stable ability in terms of MCC value among five features. MLP outperformed other single classifiers in RPSSM features according to the MCC value. In all cases, the highest MCC value was 0.763 when the RPSSM feature was used for training in MLP. It provided more extensive and persuasive evidence for various performances with various features and classifiers. It is unreliable only to use one feature and a single model to identify Acrs protein.

**Fig. 2:**
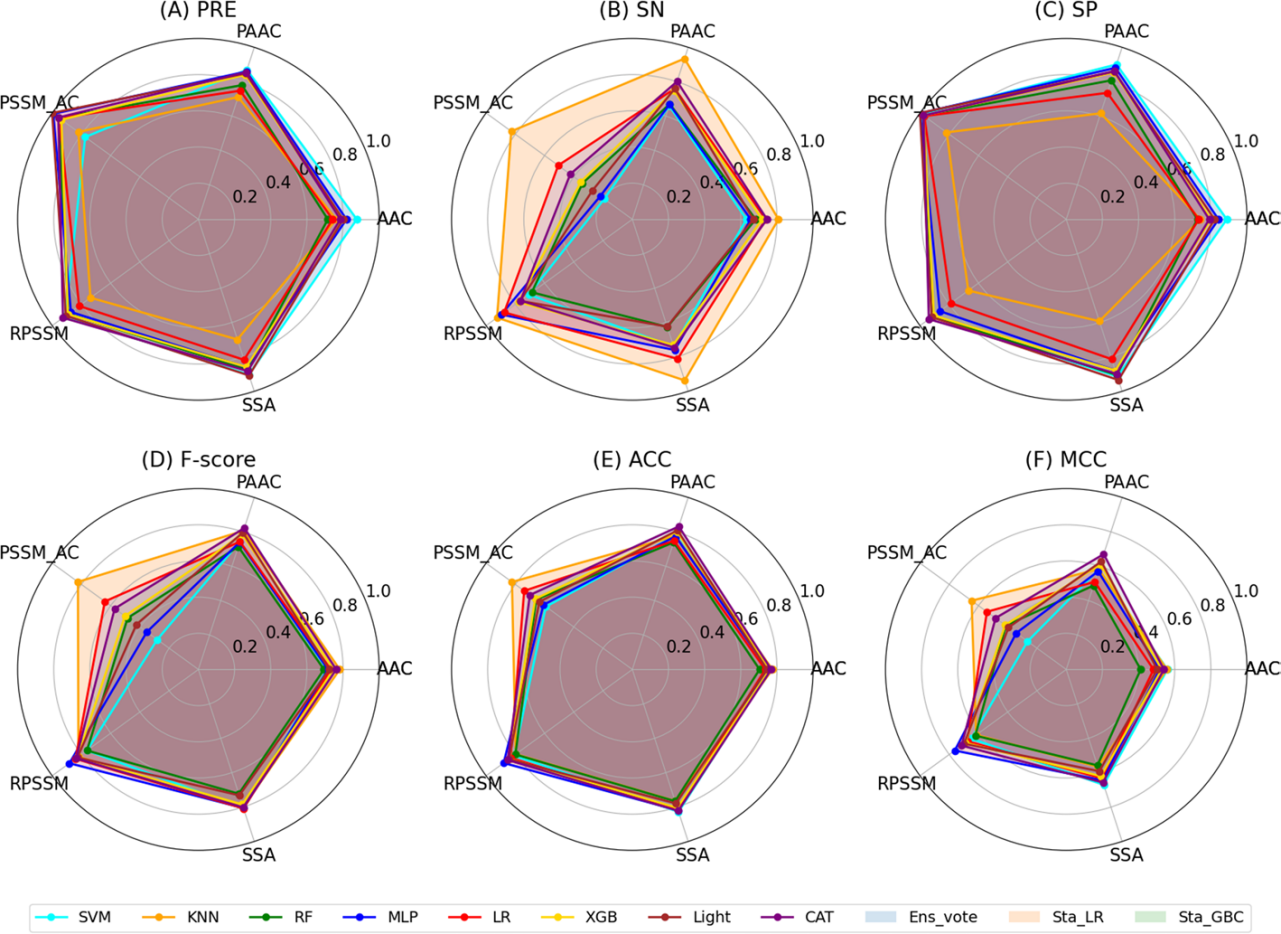
The six matrices PRE, SP, SN, F-score, ACC and MCC values of various classifiers in five types of encoding features based on five-fold cross-validation

Although some single classifiers have shown good performance for predicting Acrs, only one classifier might not be robust and reliable enough. In order to build a more comprehensive, reliable, and robust predictor, three ensemble methods have been adopted based on eight single classifiers in this study. Three ensemble methods integrated other classifiers by three different principles. Table 1 and Fig. 2 illustrate that three ensemble methods achieved better performance than single classifiers in terms of AUC value in most features, demonstrating the superiority of ensemble learning. This observation is supported by the study of Zou et al. [24].

### Performance evaluation of various ensemble features

As we mentioned above, five features were trained by eight different classifiers, respectively. Since single features cannot comprehensively represent the Acrs for identification, we attempted to integrate five single features in two ways: ensemble feature and combination feature. For combination features, we combined singles features into a vector to train models [25–27]. We explored the contribution of a variety of combined features to the prediction models of Acrs (Additional file 1: Table S1). For ensemble features, first, we trained eight different classifiers (including ensemble classifier) with five single features, then integrated classifiers of five features as an ensemble model. This study discussed ensemble features detailly because they showed better performance than combination features. For every single feature in each classifier, we have obtained its probability score of Acrs. The output of two-feature ensemble models is obtained by averaging the predictive scores of two single features in the same model. For example, we averaged the predictive scores of predicted Acrs obtained by the AAC feature trained in the SVM model and the PAAC feature trained in the same model, and we labeled it as ‘AAC&PAAC’. Therefore, the three-feature ensemble models were obtained by averaging the predictive scores of three single features in the same model, and *Feature1&Feature2&Feature3* represented the three-ensemble features*.* The four-ensemble features and the five-ensemble feature were also shown similarly. Finally, we used the averaged predictive scores as the final scores of the ensemble feature in every classifier. From the cross-validation results, the ensemble features achieved good performance for Acrs identification. By comparing the performance of all ensemble features, the ensemble feature PSSM_AC&RPSSM&SSA showed the best performance with the highest AUC value. The second-best ensemble feature is PAAC&PSSM_AC&RPSSM, and the PSSM_AC&RPSSM ensemble feature is the third best. We found that all the top 12 ensemble features include the RPSSM encoding method from Additional file 2: Table S2. These observations also demonstrated that the RPSSM feature plays an essential role in Acrs prediction.

### Performance evaluation of ensemble learning model

In the above section, ensemble classifiers with five single features have shown an excellent ability to predict Acrs, and the Sta-LR method obtained the best performance in terms of metrics. Therefore, we used the Sta-LR classifier to train various features in this study. Besides, we compared combination features with ensemble features in the same model. The ensemble feature achieved superior performance than combination features in most classifiers. Among all models, the average AUC value of Sta-LR classifiers using PSSM_AC, RPSSM and SSA features (the three-ensemble feature PSSM_AC&RPSSM&SSA) achieved the highest 0.969. Besides, the Sta-LR classifier with PSSM_AC&RPSSM&SSA ensemble feature achieved an excellent performance in terms of a high PRE value of 0.978, a high MCC value of 0.754, an ACC value of 0.866 and an F-score of 0.848 based on the fivefold cross-validation test. Based on these findings, we constructed a PreAcrs predictor to predict Acrs with a default setting: eight machine learning classifiers (SVM, KNN, MLP, LR, RF, XGBoost, LightGBM, CatBoost) were integrated into an ensemble classifier (Sta-LR); three features PAAC_AC, RPSSM, and SSA were trained by the Sta-LR classifier, separately, and three models could be obtained in this step. Then, we could obtain the PreAcrs predictor by averaging the score of the three models. The PreAcrs predictor achieved a stable and accurate prediction performance in the fivefold cross-validation and independent dataset.

### Performance comparison with other existing methods

In order to further evaluate the performance of the PreAcrs predictor, we compared PreAcrs with the state-of-the-art Acrs predictor PaCRISPR. This machine learning model was proposed by Wang et al. [19], and significantly outperformed other methods such as AcRanker and BLAST on their independent dataset. Four evolutionary features, PSSM-composition, DPC PSSM, PSSM_AC and RPSSM, were adopted in the PaCRISPR predictor, which was constructed by 10 SVM classifiers. Besides, the BLAST-based predictor, AcRanker and the hidden Markov model (HMM) based predictor were implemented for the comparison. For the BLAST-based predictor, each protein in the independent dataset was searched against all samples in the training dataset based on BLAST + software [28] and was predicted as Acr when it has the highest similarity with positive samples. The predicted results of the other three predictors could be obtained from the webserver (https://pacrispr.erc.monash.edu/AcrHub).

**Fig. 3:**
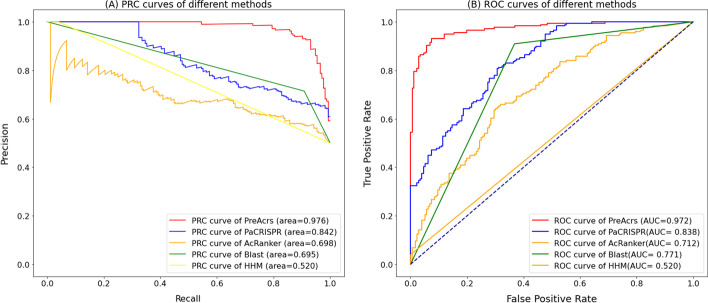
The precision-recall curves (**A**) and the ROC curves (**B**) are produced by the two existing state-of-the-art methods and the *PreAcrs* in the independent dataset

Figures 3 show that the performance of PreAcrs is better than the other predictors on the independent dataset based on the AUC and AUPRC values. The performance demonstrates that the PreAcrs method is more suitable for capturing the intrinsic patterns of non-homologous Acrs than other predictors. From other metrics (Table 2), HMM obtained higher PRE and SP values than PreAcrs, but it does not indicate that HMM outperformed PreAcrs. It means the false positive is lower and one possible reason for it is HMM prone to predict the queried proteins as non-Acrs. HMM uses probabilistic models to search homologous protein sequences. The homology-based baseline predictors made a biased prediction, as HMM failed to recognize Acrs. It predicted the Acrs with extremely high accuracy (the lowest FP) but classified many true Acrs into non-Acrs (the highest FN). HMM obtained the best PRE with the cost of predicting most Arcs as non-Acrs. This observation is supported by the work of Wang et al. [19]. Therefore, when considering the FN and FP, HMM showed poor performance when it was evaluated. According to other more critical metrics like ACC, F-score and MCC, PreAcrs outperformed the other four approaches.

**Table 2:** Performance comparison between PreAcrs and existing methods based on the independent test

Method	PRE	Sensitivity	Specificity	F-score	ACC	MCC	AUC	AUPRC
BLAST	0.714	0.910	0.632	0.800	0.771	0.564	0.771	0.695
PaCRISPR	0.816	0.528	0.881	0.641	0.705	0.437	0.838	0.842
AcRanker	0.692	0.409	0.818	0.514	0.614	0.249	0.712	0.698
HMM	**1.000**	0.039	**1.000**	0.076	0.5200	0.142	0.520	0.520
PreAcrs	0.986	**0.795**	0.989	**0.881**	**0.892**	**0.799**	**0.972**	**0.976**

The bold values indicate the best performance

We listed the predictive scores of five experimentally validated Acrs on the independent test as a case study to further evaluate the performance of PreAcrs (Table 3). The PreAcrs achieved better performance than PaCRISPR and AcRanker. For the AcrIIA7 and AcrIIA9, PaCRISPR predicted lower scores, and the predictive score of AcrIIA7 was 0.407. In contrast, PreAcrs gave these three Acrs higher scores. For AcrIIC2, PaCRISPR showed better performance, but PreAcrs also gave considerable scores. PaCRISPR only considered four features driven from evolution information and the SVM model, while PreAcrs incorporated the SSA feature from the pre-trained model and eight different models. Considering more information and various classifiers, PreAcrs showed a more robust and accurate prediction performance.

**Table 3:** The predictive scores of the case study Acrs

Acrs	PaCRISPR	AcRanker*	PreAcrs
AcrIIA7-980	0.407	− 5.949	0.800
AcrIIA9-1120	0.503	− 5.494	0.857
AcrIIA9-1158	0.531	− 5.242	0.791
AcrIIC2-DAW	0.791	− 5.266	0.746
AcrIIC2-DAS	0.833	− 5.379	0.744

^*^The threshold of AcRanker is − 5

## Conclusions

The identification of candidate Acrs plays a vital role in manipulating CRISPR-Cas machinery as a tool in gene editing or gene therapy. Using the machine learning method to identify the new Acrs based on the protein sequence can accelerate the discovery of Acrs. In this work, we proposed a machine learning-based ensemble framework, PreAcrs, to accurately and efficiently identify Acrs from protein sequences. PreAcrs extracted distinctive characteristics from experimentally validated Acrs by combining the evolutionary features with the pretrained model feature with multiple models. The features were trained by an ensemble classifier constructed by eight base classifiers. PreAcrs predictor displayed a good performance for predicting new Acrs in terms of prediction accuracy and robustness. We anticipate that PreAcrs will be extensively used in Acrs prediction and help researchers to have a comprehension understanding of Acrs. PreAcrs shows excellent performance compared to the existing methods, but it still has some limitations. One limitation is that only the mRMR algorithm is applied to select significant features in PreAcrs, so some biases in this step may reduce the predictive accuracy. Another limitation is that PreAcrs does not provide a visual and user-friendly website; it may be difficult for some biologists to analyze Acrs. In future works, we may use multiple feature selection algorithms to calculate feature importance to obtain a reasonable feature, and build a powerful, user-friendly and interactive website.

## Methods and materials

### Overall framework of PreAcrs

**Fig. 4:**
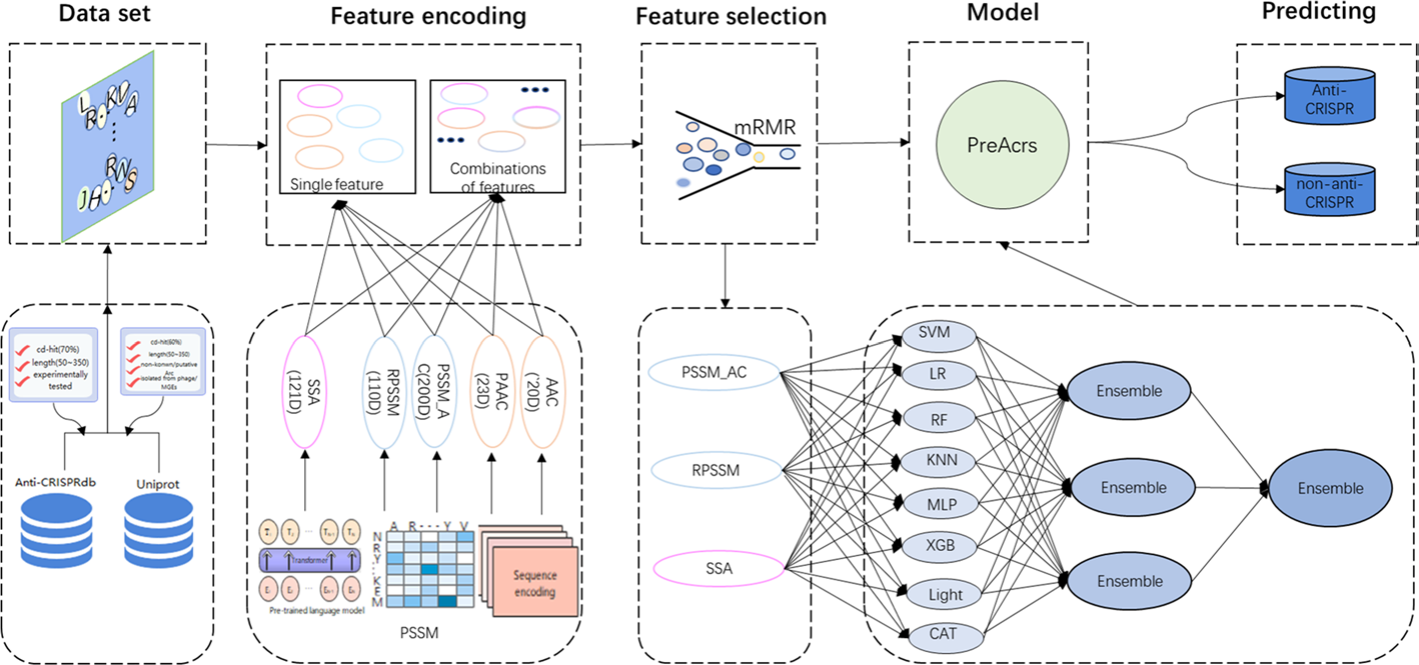
The flowchart of the *PreAcrs* framework for Acrs prediction. The five major steps for constructing *PreAcrs* include data collection, feature encoding, feature selection, model construction, and performance evaluation

Figure 4 shows the overall workflow of the PreAcrs framework, including five major steps: Dataset collection and curation, Feature encoding, Feature selection, Model training, and Model validation. These steps are described in the following sections.

### Dataset collection and curation

To build a powerful Acrs predictive model, we need to construct a training dataset and an independent test dataset comprised of two parts: positive samples (experimentally validated Acrs) and negative samples (non-Acrs). As mentioned above, Anti-CRISPRdb, AcrDB, and AcrCatalog are online databases of anti-CRISPR proteins. The latest update time of the Anti-CRISPRdb database is January 2021, and it has 1378 experimentally validated entries.

The AcrDB and AcrCatalog are databases of computationally predicted Acrs. In this study, we collected the experimentally validated Acrs from Anti-CRISPRdb, which is the latest database and contains more experimentally validated Acrs than others. We extracted 1,378 experimentally validated Acrs from the Anti-CRISPRdb [12] and 17 newly discovered experimentally validated Acrs from NCBI. To construct a robust machine learning model and eliminate the redundant Arcs, we used CD-HIT [29] to remove the highly-homologous sequences. Here, we set the identification threshold as 70% in CD-HIT (removed those sequences with more than 70% similarity). 588 Acrs sequences were obtained, and their length ranges from 50 to 350. After the 588 Acrs were randomly divided into two parts with a ratio of 7:3, we obtained 412 Acrs in the training dataset and 176 Acrs in the independent dataset.

Because there is no standard set of non-Arcs, constructing a comprehensive and reasonable non-Acrs dataset is a challenging and vital question. In this study, we referred to the work of Wang et al. [19] to construct the non-Acrs dataset. Because the range of Acrs sequence length is fixed, and most Acrs were found from a limited set of phages and mobile genetic elements (MGEs), the negative samples were selected with four strict criteria from Uniprot. The four criteria are the following: (1) must not be known or putative Acrs; (2) must be isolated from phage or bacterial MGEs (known or putative MEGs); (3) must have < 40% sequence similarity to each other and the 588 positive samples; (4) the lengths must fall in the range between 50 and 350 residues. According to the above four criteria, 1571 non-Acrs were obtained in this study. Then, we randomly selected 412 non-Acrs as negative samples in the training dataset and 176 non-Acrs as negative samples in the independent dataset. Each negative sample was only included in one dataset. In this way, the training dataset has 412 positive and 412 negative samples, while the independent test dataset contains 176 positive and 176 negative samples (Table 4). In addition, we chose 5 Acrs from the independent dataset as a case study.

**Table 4:** The statistics of datasets employed in this study

	Number of anti-CRISPR proteins	Number of non-anti-CRISPR proteins	Total
Training dataset	412	412	824
Independent test dataset	176	176	352
Total	588	588	1176

### Feature encoding

In order to find the features that could better represent Acrs, we firstly evaluated 18 types of features to represent Acrs, including the composition of *k*-spaced amino acid pairs (CKSAAP), amino acid composition (AAC), pseudo amino acid composition (PAAC), bidirectional long short-term memory (BiLSTM), soft sequence alignment (SSA), PSSM_AC, RPSSM and PSSM-composition et. (Table 5 and Additional file 3: Table S3). We selected five features (AAC, PAAC, PSSM_AC, RPSSM, SSA) considering the computational requirements and predictive performance. The five features could be categorized into three groups: sequence features, evolutionary features, and pre-trained model features. These features have been widely applied in feature encoding research [19, 30, 31] and have achieved a good performance in protein properties and function predictions [32–38]. The following are the five features adopted in this study.

**Table 5:** Features: the sequence and structural features calculated and their dimensionalities

Feature type	Feature cluster	Dimensions	Reduced-dimensions
Sequence	AAC	20	20
	PAAC	23	23
	CKSAAP	2400	200
	DDE	400	200
	DPC	400	200
Evolutionary	PSSM-composition	400	200
	DPC-PSSM	400	200
	PSSM-AC	200	200
	RPSSM	110	110
	PSSM-SMTH	1000	200
Pre-trained	BiLSTM	3605	200
	LM	533	200
	SSA	121	121
	TAPE-BERT	768	200
	UniRep	1900	200
	W2V	300	200
	esm	1280	200
	ProtTrans	1024	200
Total		14,884	3074

#### AAC

As one of the most important features, amino acid composition (AAC) has been successfully applied in many bioinformatics fields, for example, protein structure classification [30], thermophilic proteins prediction [39], and protein–protein interactions identification [40]. For AAC, each sequence is represented by a 20-dimensional numerical vector, in which each number corresponds to the frequency of an amino acid type in the whole protein sequence [41]. Every element in AAC of a given protein $\mathrm{P}$ could be calculated by the following formula:

$$P=\left[\begin{array}{c}{p}_{1}\\ {p}_{2}\\ \begin{array}{c}\vdots \\ {p}_{20}\end{array}\end{array}\right]$$

with

$${p}_{i}=\frac{{c}_{i}}{L}, (i=1, 2, \cdots , 20)$$

where ${c}_{i}$ is the number of type $i$ native amino acid in the whole protein $P$ sequence, and $L$ is the length of the protein $\mathrm{P}$ sequence. Finally, the ${p}_{i}$ is the frequency of type $i$ native amino acid in the protein $\mathrm{P}$.

#### PAAC

Pseudo-Amino acid composition (PAAC) was proposed by Zhou [42] for predicting cellular protein attributes and has been widely used in many studies [31, 43]. This group of descriptors involves sequence-order information, hydrophobicity value, hydrophilicity value, and side-chain mass. The PAAC is defined by 20 + λ discrete numbers:

$$\mathrm{P}=\left[{p}_{1},p,\dots ,{p}_{20+1},\dots ,{p}_{20+\uplambda }\right]$$

with

$${p}_{c}=\frac{{f}_{c}}{\sum_{c=1}^{20}{f}_{c}+\omega \sum_{j=1}^{\lambda }{\theta }_{j}}, \quad (1<c<20)$$

$${p}_{c}=\frac{\omega {\theta }_{c-20}}{\sum_{c=1}^{20}{f}_{c}+\omega \sum_{j=1}^{\lambda }{\theta }_{j}},\quad (21<c<20+\lambda )$$

$${\theta }_{j}=\frac{1}{L-\lambda }\sum_{i=1}^{N-1}\Theta (P({S}_{i}),P({S}_{i+j}))$$

where the ${f}_{c}$ is the normalized frequency of amino acid $\mathrm{c}$ in the protein sequence. L is the length of protein and θ_j_ is the jth rank of the coupling factor*.* $\Theta (P({S}_{i}),P({S}_{i+j}))$ represents the correlation function, and λ is the maximum correlation length. This study used iLearnPlus to extract PAAC feature-based protein sequences [44] and generated a 23-dimensional feature vector for each protein.

#### PSSM-AC

PSSM-AC is derived from Position-Specific Scoring Matrix (PSSM) by applying the auto covariance (AC) transformation to each column of PSSM, and it measures the average correlation between two elements within the PSSM [45, 46]. A 20 × *G*-dimensional vector represents each sequence in PSSM-AC by the following formula:

$$PSSM-AC(j,g)=\frac{1}{L-g}\sum_{i=1}^{L-g}{(P}_{i,j}-\overline{{P }_{j}})\times ({P}_{i+g,j}-\overline{{P }_{j}})$$

with

$$\overline{{P }_{j}}=\frac{1}{L}\sum_{i=1}^{L}{P}_{i,j},\quad (j=1, 2, 3, \cdots , 20)$$

where ${P}_{i,j}$ represents the PSSM value at the $\mathrm{ith}$ row and jith column, and the $\overline{{P }_{j}}$ is the average value of amino acid j in the whole protein sequence. $\mathrm{G}$ is a number smaller than the length of the whole protein sequence L, and the $\mathrm{g}$ ranges from 1, 2, …, G; here, $\mathrm{G}$ is set to 10 in this study [47]. Therefore, a 200-dimensional feature vector is generated for each protein.

#### RPSSM

According to the work of Li et al. [48], the original PSSM profile (L × 20) could be reduced to a L × 10 matrix by merging some columns. RPSSM is obtained by exploring the local sequence information based on the L × 10 reduced PSSM [49, 50]:

$$re-PSSM=({P}_{1}, {P}_{2},{P}_{3}, \cdots , {P}_{10})$$

and

$${P}_{1}=\frac{{p}_{F}+{p}_{Y}+{p}_{W}}{3}, {P}_{2}=\frac{{p}_{M}+{p}_{L}}{2}, {P}_{3}=\frac{{p}_{I}+{p}_{V}}{2}, {P}_{4}=\frac{{p}_{A}+{p}_{T}+{p}_{S}}{3}$$

$${P}_{5}\frac{{p}_{N}+{p}_{H}}{2}, {P}_{6}=\frac{{p}_{Q}+{p}_{E}+{p}_{D}}{3}, {P}_{7}=\frac{{p}_{R}+{p}_{K}}{2}, {P}_{8}={p}_{C}, {P}_{9}={p}_{G}, {P}_{10}={p}_{P}$$

where $p_{A} ,p_{R} , \ldots ,p_{V}$ represent the 20 columns in the original PSSM profile corresponding to the 20 amino acids. The re-PSSM is further transformed into a 10-dimensional vector:

$${E}_{j}=\frac{1}{L}{\sum_{i=1}^{L}({p}_{i,j}-{\overline{p} }_{j})}^{2}$$

and

$$\overline{{p }_{j}}=\frac{1}{L}\sum_{i=1}^{L}{p}_{i,j}, (j=1, 2, \cdots , 10; i=1, 2, \cdots , L)$$

Additionally, the re-PSSM can be further transformed into a 10 × 10 matrix to capture the local sequence-order information by this formula:

$${E}_{j, t}=\frac{1}{L-1}\sum_{i=1}^{L-1}\frac{{({p}_{i, j}-{p}_{i+1,t})}^{2}}{2}, (s,t=1, 2, 3,\cdots , 10)$$

where ${p}_{i,j}$ represents the element at the ith row and jth column of there-PSSM. Finally, a 110-dimensional RPSSM feature is obtained by combining ${E}_{j,t}$ and ${E}_{j}$:

$$RPSSM=[{E}_{\mathrm{1,1}},{E}_{\mathrm{1,2}},\cdots ,{E}_{\mathrm{10,10}},{E}_{1},\cdots ,{E}_{10}]$$

#### Pretrained SSA embedding

The pretrained SSA embedding mosdel is obtained by combining the pre-trained language model with the soft sequence alignment (SSA) [51]. First, an embedding matrix R^L×121^ is given using the stacked BiLSTM encoders for each sequence, where L is the protein sequence length [52]. Then, the pretrained SSA embedding model is trained and optimized by SSA, which the following formulas could describe. For convenience, we supposed two embedding matrices P1(R^L1×121^) and P2(R^L2×121^), of two different protein sequences with lengths L_1_ and L_2_, respectively:

$${P}_{1}=[{x}_{1},{x}_{2},\cdots ,{x}_{L1}], {P}_{2}=[{y}_{1},{y}_{2},\cdots ,{y}_{L2}]$$

where x_i_, y_i_ are vectors with 121-dimension.

The following formula represents the similarity of P_1_ and P_2_:

$$\widehat{p}=-\frac{1}{A}\sum_{i=1}^{L1}\sum_{j=1}^{L2}{\alpha }_{ij}\Vert {x}_{i}-{{y}_{j}\Vert }_{1}$$

and

$$A=\sum_{i=1}^{L1}\sum_{j=1}^{L2}{\alpha }_{ij}, { \alpha }_{ij}={\delta }_{ij}+{\varepsilon }_{ij}-{\delta }_{ij}{\varepsilon }_{ij}$$

with

$${\delta }_{ij}=\frac{exp(-\Vert {x}_{i}-{{y}_{k}\Vert }_{1})}{\sum_{k=1}^{L2}exp(-\Vert {x}_{i}-{{y}_{k}\Vert }_{1})}, {\varepsilon }_{ij}=\frac{exp(-\Vert {x}_{k}-{{y}_{j}\Vert }_{1})}{\sum_{k=1}^{L1}exp(-\Vert {x}_{k}-{{y}_{j}\Vert }_{1})}$$

The SSA embedding model could convert each protein sequence into an embedded matrix R^L×121^, and finally, an average pooling operation obtained a 121-dimensional feature.

### Feature selection

Original features are represented by a high dimensional vector or matrix, which would raise severe problems in machine learning algorithms, such as overfitting, time-consuming training process and high requirement of computing resources. Therefore, identifying the most contributing information and features plays a vital role in performance improvement. As one of the most popular feature selection algorithms, maximum relevance minimum redundancy (mRMR) was proposed by Peng et al. [53] and has been applied in many studies and achieved robust performances [54–56]. In this study, mRMR was used to identify the most important features and improve the generalization ability of the model.

### Machine learning algorithm

In this study, we focused on the traditional machine learning classification methods, including support vector machine, k-nearest neighbor, multi-layer perceptron, logistic regression, random forest, extreme gradient boosting, Light gradient boost machine and ensemble method that integrates the previous eight classification methods by hard voting strategy and stacking classifiers. More information is shown in the following subsections.

#### Support vector machine

Support vector machine (SVM) was first proposed by Vapnik et al. [57], and has successfully dealt with some binary classification problems in bioinformatics [25, 58, 59]. Two parameters Cost (*C*) and Gamma (*γ*) affect the performance of the SVM model with the RBF kernel. In this study, we used the grid search strategy to optimize *C* and *γ* in the space {2^−6^, 2^−5^, …, 2^5^, 2^6^}. Finally, an SVM classifier with the optimal value of *C* and *γ* was constructed.

#### K-nearest neighbor

K-nearest neighbor (KNN) is a fundamental classifier that has been applied in predicting protein function [60], extracting protein–protein information [61], and predicting eukaryotic protein subcellular [62]. The performance of KNN is directly affected by the parameter *k*. In this study, a grid search within the space $\left\{ {1,2, \ldots ,\max \left\{ {\sqrt {FeaNum} ,\frac{FeaNum}{2}} \right\}} \right\}$ was applied to optimize the parameter k during model training, where *FeaNum* is the number of features used in modelling.

#### Multi-layer perception

Multi-layer perceptron (MLP) is known as a type of artificial neural network (ANN) [63, 64]. MLP has been applied in many bioinformatics studies, such as the prediction of protein structure classes [65], protein tertiary structure [66], and DNA–protein binding sites [67]. In this study, an MLP classifier with two hidden layers was trained, and the first and second hidden layers have 64 and 32 nodes, respectively. The maximum learning iterations is 1000.

#### Logistic regression

Logistic regression (LR) is widely used to predict the probability of an event happening [59, 68], which the following formula could represent:

$$p(y)=\frac{1}{1+{e}^{-({\beta }_{0}+{\beta }_{1}\chi )}}$$

where *p*(*y*) is the expected probability of dependent variable $\mathrm{y}$, and *β*_0_ and *β*_1_ are constants.

#### Random forest

Random forest (RF) classifier is proposed by Breiman [69] and has been used in the prediction of type IV secreted effector proteins [70] and protein structural class [59]. To find the optimal number of the trees M and features mtry, we used a gird searching to optimize $\mathrm{M}$ and $\mathrm{mtry}$ within space $\{1, 2,\cdots ,\mathrm{max}\left\{\sqrt{FeaNum},\frac{FeaNum}{2}\right\}\}$ and {1, 6, 11, 16}, respectively, where *FeaNum* is the number of features adopted during modeling.

#### XGBoost

Extreme gradient boosting (XGBoost) is a scalable end-to-end tree boosting system [71] and has been widely used as a fast and highly effective machine learning method [72, 73]. Eitzinger et al. implemented AcRanker using XGBoost to identify Acrs [14, 16]. In this study, the default parameters are adopted in the XGBoost model, except for the learning rate of 0.1.

#### LightGBM

Light gradient boost machine (LightGBM) shows excellent performance when the feature dimension is high and the larger data size [21]. LightGBM has been used in identifying miRNA targets [74] and predicting the protein–protein interactions [75] and the blood–brain-barrier penetration [76]. This study used the *LightGBM* package with default parameters in python during experiments.

#### CatBoost

CatBoost achieves state-of-the-art results since it successfully handles categorical features and calculates leaf values via a new scheme, which helps reduce overfitting [23]. Catboost has been applied in various tasks, including molecular structure relationship and the biological activity prediction [77] and the identification of pyroptosis-related molecular subtypes of lung adenocarcinoma [78]. In this study, the parameters of CatBoost were set as default values.

#### Ensemble learning method

This study proposed three ensemble models to construct more robust and reliable classifiers, which predicted new Acrs proteins by integrating the above eight classifiers (SVM, KNN, MLP, LR, RF, XGB, LightGBM, and CatBoost) through the hard voting rule (Ens-vote) or two stacking classifiers with logistic regression (Sta-LR) and gradient boosting classifier (Sta-GBC) [79], respectively.

### Performance assessment

Fairly evaluating the classification methods' predictive performance is an essential subject in machine learning. In this study, we used six measurements, namely, Sensitivity (SN), Specificity (SP), Accuracy (ACC), Precision (PRE), F1-score, and Matthew’s correlation coefficient (MCC) [80], which are denoted as:

$$SN=\frac{TP}{TP+FN}$$

$$SP=\frac{TN}{TN+FP}$$

$$PRE=\frac{TP}{TP+FP}$$

$$ACC=\frac{TP+TN}{TP+FP+TN+FN}$$

$$F-score=2\times \frac{TP}{2TP+FP+FN}$$

$$\mathrm{MCC}=\frac{TP\times TN-FP\times FN}{\sqrt{\left(TP+FN\right)\times \left(TN+FP\right)\times \left(TP+FP\right)\times \left(TN+FN\right)}}$$

where TP, TN, FP, and FN are the number of true positive, true negative, false positive and false negative, respectively. Besides, the area under the receiver operating characteristic (ROC) curve (AUC) is also used to assess the performance, and the ROC was shown in a plot of the TP rate versus the FP rate. All methods were evaluated based on a fivefold cross-validation.

## Supplementary Information

**Additional file 1: Table S1.** Performance of all single features.

**Additional file 1: Table S2.** Performance of ensemble features.

**Additional file 1: Table S3.** Performance of combinational features.

## Data Availability

The datasets of this study are available on Github (https://github.com/Lyn-666/anti_CRISPR.git).

## Abbreviations

Acrs: Anti-CRISPR proteins
AAC: Amino acid composition
PAAC: Pseudo-amino acid composition
PSSM: Position specific scoring matrix
PSSM-AC: Position-specific matrix auto covariance
RPSSM: Reduced position specific scoring matrix
SSA: Soft sequence alignment
mRMR: Maximum relevance minimum redundancy
SVM: Support vector machine
KNN: K-nearest neighbor
MLP: Multi-layer perceptron
LR: Logistic regression
RF: Random forest
XGBoost: Extreme gradient boosting
LightGBM: Light gradient boost machine
SN: Sensitivity
SP: Specificity
PRE: Precision
TP: True positive
FP: False positive
TN: True negative
FN: False negative
MCC: Matthews correlation coefficient
AUC: Area under the ROC curve
ACC: Accuracy
PRC: Precision-recall curve
ROC: Receiver operating characteristic
AUPRC: Area under the PRC

## References

[1] Barrangou R, Fremaux C, Deveau H, Richards M, Boyaval P, Moineau S, Romero DA, Horvath P (2007). CRISPR provides acquired resistance against viruses in prokaryotes. Science.

[2] Marraffini LA, Sontheimer EJ (2008). CRISPR interference limits horizontal gene transfer in staphylococci by targeting DNA. Science.

[3] Bondy-Denomy J, Pawluk A, Maxwell KL, Davidson AR (2013). Bacteriophage genes that inactivate the CRISPR/Cas bacterial immune system. Nature.

[4] Pawluk A, Davidson AR, Maxwell KL (2018). Anti-CRISPR: discovery, mechanism and function. Nat Rev Microbiol.

[5] Stanley SY, Maxwell KL (2018). Phage-encoded anti-CRISPR defenses. Annu Rev Genet.

[6] Marino ND, Zhang JY, Borges AL, Sousa AA, Leon LM, Rauch BJ, Walton RT, Berry JD, Joung JK, Kleinstiver BP (2018). Discovery of widespread type I and type V CRISPR-Cas inhibitors. Science.

[7] Watters KE, Fellmann C, Bai HB, Ren SM, Doudna JA (2018). Systematic discovery of natural CRISPR-Cas12a inhibitors. Science.

[8] Pawluk A, Staals RH, Taylor C, Watson BN, Saha S, Fineran PC, Maxwell KL, Davidson AR (2016). Inactivation of CRISPR-Cas systems by anti-CRISPR proteins in diverse bacterial species. Nat Microbiol.

[9] Uribe RV, Van Der Helm E, Misiakou M-A, Lee S-W, Kol S, Sommer MOA (2019). Discovery and characterization of Cas9 inhibitors disseminated across seven bacterial phyla. Cell Host Microbe.

[10] Forsberg KJ, Bhatt IV, Schmidtke DT, Javanmardi K, Dillard KE, Stoddard BL, Finkelstein IJ, Kaiser BK, Malik HS (2019). Functional metagenomics-guided discovery of potent Cas9 inhibitors in the human microbiome. Elife.

[11] Pawluk A, Amrani N, Zhang Y, Garcia B, Hidalgo-Reyes Y, Lee J, Edraki A, Shah M, Sontheimer EJ, Maxwell KL (2016). Naturally occurring off-switches for CRISPR-Cas9. Cell.

[12] Dong C, Hao G-F, Hua H-L, Liu S, Labena AA, Chai G, Huang J, Rao N, Guo F-B (2018). Anti-CRISPRdb: a comprehensive online resource for anti-CRISPR proteins. Nucleic Acids Res.

[13] Wang J, Dai W, Li J, Li Q, Xie R, Zhang Y, Stubenrauch C, Lithgow T (2020). AcrHub: an integrative hub for investigating, predicting and mapping anti-CRISPR proteins. Nucleic Acids Res.

[14] Huang L, Yang B, Yi H, Asif A, Wang J, Lithgow T, Zhang H, Minhas A, Ul Amir F, Yanbin Y (2021). AcrDB: a database of anti-CRISPR operons in prokaryotes and viruses. Nucleic Acids Re.

[15] Zhang F, Zhao S, Ren C, Zhu Y, Zhou H, Lai Y, Zhou F, Jia Y, Zheng K, Huang Z (2018). CRISPRminer is a knowledge base for exploring CRISPR-Cas systems in microbe and phage interactions. Commun Biol.

[16] Eitzinger S, Asif A, Watters KE, Iavarone AT, Knott GJ, Doudna JA, Minhas A, Ul Amir F (2020). Machine learning predicts new anti-CRISPR proteins. Nucleic Acids Res.

[17] Yi H, Huang L, Yang B, Gomez J, Zhang H, Yin Y (2020). AcrFinder: genome mining anti-CRISPR operons in prokaryotes and their viruses. Nucleic Acids Res.

[18] Gussow AB, Shmakov SA, Makarova KS, Wolf YI, Bondy-Denomy J, Koonin EV (2020). Vast diversity of anti-CRISPR proteins predicted with a machine-learning approach.

[19] Wang J, Dai W, Li J, Xie R, Dunstan RA, Stubenrauch C, Zhang Y, Lithgow T (2020). PaCRISPR: a server for predicting and visualizing anti-CRISPR proteins. Nucleic Acids Res.

[20] Gussow AB, Park AE, Borges AL, Shmakov SA, Makarova KS, Wolf YI, Bondy-Denomy J, Koonin EV (2020). Machine-learning approach expands the repertoire of anti-CRISPR protein families. Nat Commun.

[21] Fernández-Delgado M, Cernadas E, Barro S, Amorim D (2014). Do we need hundreds of classifiers to solve real world classification problems?. J Mach Learn Res.

[22] Ke G, Meng Q, Finley T, Wang T, Chen W, Ma W, Ye Q, Liu T-Y. Lightgbm: a highly efficient gradient boosting decision tree. In: Advances in neural information processing systems. 2017, p. 30.

[23] Dorogush AV, Ershov V, Gulin A. CatBoost: gradient boosting with categorical features support 2018. arXiv preprint https://arxiv.org/abs/1810.11363.

[24] Zou L, Chen K. Computational prediction of bacterial type IV-B effectors using C-terminal signals and machine learning algorithms. In: 2016 IEEE conference on computational intelligence in bioinformatics and computational biology (CIBCB). IEEE;2016.

[25] Zou L, Nan C, Hu F (2013). Accurate prediction of bacterial type IV secreted effectors using amino acid composition and PSSM profiles. Bioinformatics.

[26] Wang Y, Wei X, Bao H, Liu S-L (2014). Prediction of bacterial type IV secreted effectors by C-terminal features. BMC Genom.

[27] Chen Z, Zhou Y, Song J, Zhang Z (2013). hCKSAAP_UbSite: improved prediction of human ubiquitination sites by exploiting amino acid pattern and properties. Biochim Biophys Acta BBA Proteins Proteom.

[28] Camacho C, Coulouris G, Avagyan V, Ma N, Papadopoulos J, Bealer K, Madden TL (2009). BLAST+: architecture and applications. BMC Bioinform.

[29] Huang Y, Niu B, Gao Y, Fu L, Li W (2010). CD-HIT Suite: a web server for clustering and comparing biological sequences. Bioinformatics.

[30] Isik Z, Yanikoglu B, Sezerman U, Aykanat C, Dayar T, Körpeoğlu İ (2004). Protein structural class determination using support vector machines. Computer and information sciences—ISCIS 2004.

[31] Chou K-C (2009). Pseudo amino acid composition and its applications in bioinformatics, proteomics and system biology. Curr Proteom.

[32] Bernardes J (2013). A review of protein function prediction under machine learning perspective. Recent Patents Biotechnol.

[33] Li F, Li C, Marquez-Lago TT, Leier A, Akutsu T, Purcell AW, Ian Smith A, Lithgow T, Daly RJ, Song J (2018). *Quokka*: a comprehensive tool for rapid and accurate prediction of kinase family-specific phosphorylation sites in the human proteome. Bioinformatics.

[34] Li F, Chen J, Leier A, Marquez-Lago T, Liu Q, Wang Y, Revote J, Smith AI, Akutsu T, Webb GI (2020). DeepCleave: a deep learning predictor for caspase and matrix metalloprotease substrates and cleavage sites. Bioinformatics.

[35] Li F, Leier A, Liu Q, Wang Y, Xiang D, Akutsu T, Webb GI, Smith AI, Marquez-Lago T, Li J (2020). Procleave: predicting protease-specific substrate cleavage sites by combining sequence and structural information. Genom Proteom Bioinform.

[36] Mei S, Li F, Xiang D, Ayala R, Faridi P, Webb GI, Illing PT, Rossjohn J, Akutsu T, Croft NP (2021). Anthem: a user customised tool for fast and accurate prediction of binding between peptides and HLA class I molecules. Brief Bioinform.

[37] Wang X, Li F, Xu J, Rong J, Webb GI, Ge Z, Li J, Song J (2022). ASPIRER: a new computational approach for identifying non-classical secreted proteins based on deep learning. Brief Bioinform.

[38] Li F, Guo X, Xiang D, Pitt ME, Bainomugisa A, Coin LJ (2022). Computational analysis and prediction of PE_PGRS proteins using machine learning. Comput Struct Biotechnol J.

[39] Wang X-F, Gao P, Liu Y-F, Li H-F, Lu F (2020). Predicting thermophilic proteins by machine learning. Curr Bioinform.

[40] Chen H, Li F, Wang L, Jin Y, Chi C-H, Kurgan L, Song J, Shen J (2021). Systematic evaluation of machine learning methods for identifying human–pathogen protein–protein interactions. Brief Bioinform.

[41] Chou K-C, Zhang C-T (1995). Prediction of protein structural classes. Crit Rev Biochem Mol Biol.

[42] Chou KC (2001). Prediction of protein cellular attributes using pseudo-amino acid composition. Proteins Struct Funct Bioinform.

[43] Chou K-C (2005). Using amphiphilic pseudo amino acid composition to predict enzyme subfamily classes. Bioinformatics.

[44] Chen Z, Zhao P, Li C, Li F, Xiang D, Chen Y-Z, Akutsu T, Daly J, Roger WI, Geoffrey ZQ (2021). *iLearnPlus*: a comprehensive and automated machine-learning platform for nucleic acid and protein sequence analysis, prediction and visualization. Nucleic Acids Res.

[45] Wold S, Jonsson J, Sjörström M, Sandberg M, Rännar S (1993). DNA and peptide sequences and chemical processes multivariately modelled by principal component analysis and partial least-squares projections to latent structures. Anal Chim Acta.

[46] Liu T, Zheng X, Wang C, Wang J (2010). Prediction of subcellular location of apoptosis proteins using pseudo amino acid composition: an approach from auto covariance transformation. Protein Pept Lett.

[47] Wang J, Yang B, Revote J, Leier A, Marquez-Lago TT, Webb G, Song J, Chou K-C, Lithgow T (2017). POSSUM: a bioinformatics toolkit for generating numerical sequence feature descriptors based on PSSM profiles. Bioinformatics.

[48] Li T, Fan K, Wang J, Wang W (2003). Reduction of protein sequence complexity by residue grouping. Protein Eng Des Sel.

[49] Ding S, Li Y, Shi Z, Yan S (2014). A protein structural classes prediction method based on predicted secondary structure and PSI-BLAST profile. Biochimie.

[50] Ding C, Han H, Li Q, Yang X, Liu T (2021). iT3SE-PX: identification of bacterial type III secreted effectors using PSSM profiles and XGBoost feature selection. Comput Math Methods Med.

[51] Bepler T, Berger B. Learning protein sequence embeddings using information from structure. 2019. https://arxiv.org/abs/1902.08661. PMC12947628 41768824

[52] Lv Z, Cui F, Zou Q, Zhang L, Xu L (2021). Anticancer peptides prediction with deep representation learning features. Brief Bioinform.

[53] Peng H, Long F, Ding C (2005). Feature selection based on mutual information criteria of max-dependency, max-relevance, and min-redundancy. IEEE Trans Pattern Anal Mach Intell.

[54] Li W, Lin K, Feng K, Cai Y (2008). Prediction of protein structural classes using hybrid properties. Mol Divers.

[55] Ni Q, Chen L (2017). A feature and algorithm selection method for improving the prediction of protein structural class. Comb Chem High Throughput Screen.

[56] Xu Y, Ding Y-X, Ding J, Wu L-Y, Xue Y (2016). Mal-Lys: prediction of lysine malonylation sites in proteins integrated sequence-based features with mRMR feature selection. Sci Rep.

[57] Boser BE, Guyon IM, Vapnik VN. A training algorithm for optimal margin classifiers. In: Proceedings of the fifth annual workshop on Computational learning theory—COLT '92. ACM Press; 1992.

[58] Yang ZR (2004). Biological applications of support vector machines. Brief Bioinform.

[59] Wang J, Yang B, An Y, Marquez-Lago T, Leier A, Wilksch J, Hong Q, Zhang Y, Hayashida M, Akutsu T (2019). Systematic analysis and prediction of type IV secreted effector proteins by machine learning approaches. Brief Bioinform.

[60] Lan L, Djuric N, Guo Y, Vucetic S (2013). MS-k NN: protein function prediction by integrating multiple data sources. BMC Bioinform.

[61] Li L, Jing L, Huang D. Protein-protein interaction extraction from biomedical literatures based on modified SVM-KNN. In: 2009 International conference on natural language processing and knowledge engineering. IEEE;2009.

[62] Chou K-C, Shen H-B (2006). Predicting eukaryotic protein subcellular location by fusing optimized evidence-theoretic K-nearest neighbor classifiers. J Proteome Res.

[63] Bishop CM (1995). Neural networks for pattern recognition.

[64] Tu JV (1996). Advantages and disadvantages of using artificial neural networks versus logistic regression for predicting medical outcomes. J Clin Epidemiol.

[65] Bao W, Chen Y, Wang D (2014). Prediction of protein structure classes with flexible neural tree. Bio-med Mater Eng.

[66] Shao G, Chen Y, Huang D-S, Ma J, Kang-Hyun Jo M, Gromiha M (2012). Predict the tertiary structure of protein with flexible neural tree. Intelligent Computing Theories and Applications.

[67] Zeng H, Edwards MD, Liu G, Gifford DK (2016). Convolutional neural network architectures for predicting DNA–protein binding. Bioinformatics.

[68] LaValley MP (2008). Logistic regression. Circulation.

[69] Breiman L (2001). Random Forests. Mach Learn.

[70] Wei L, Liao M, Gao X, Zou Q (2015). An improved protein structural classes prediction method by incorporating both sequence and structure information. IEEE Trans NanoBiosci.

[71] Chen T, Guestrin C. XGBoost. In: Proceedings of the 22nd ACM SIGKDD international conference on knowledge discovery and data mining. ACM; 2016.

[72] Li W, Yin Y, Quan X, Zhang H (2019). Gene expression value prediction based on XGBoost algorithm. Front Genet.

[73] Zhong J, Sun Y, Peng W, Xie M, Yang J, Tang X (2018). XGBFEMF: an XGBoost-based framework for essential protein prediction. IEEE Trans NanoBiosci.

[74] Wang D, Zhang Y, Zhao Y. LightGBM: an effective miRNA classification method in breast cancer patients. In: Proceedings of the 2017 international conference on computational biology and bioinformatics. 2017, p. 7–11.

[75] Chen C, Zhang Q, Ma Q, Yu B (2019). LightGBM-PPI: predicting protein-protein interactions through LightGBM with multi-information fusion. Chemom Intell Lab Syst.

[76] Shaker B, Yu M-S, Song JS, Ahn S, Ryu JY, Oh K-S, Na D (2021). LightBBB: computational prediction model of blood–brain-barrier penetration based on LightGBM. Bioinformatics.

[77] Hamzah H, Bustamam A, Yanuar A, Sarwinda D. Predicting the molecular structure relationship and the biological activity of dpp-4 inhibitor using deep neural network with Catboost method as feature selection. In: 2020 International conference on advanced computer science and information systems (ICACSIS). IEEE; 2020, pp. 101–108.

[78] Ping LL, Lu L, Zhao Q, Kou Q, Wu X, Jiang Z, Rong G, Luo Y, Zhao Q (2021). Identification and validation of the pyroptosis-related molecular subtypes of lung adenocarcinoma by bioinformatics and machine learning. Front Cell Dev Biol.

[79] Alexandropoulos SAN, Aridas CK, Kotsiantis SB, Vrahatis MN. Stacking strong ensembles of classifiers. In: IFIP International Conference on Artificial Intelligence Applications and Innovations. Springer, Cham. 2019; pp. 545–556.

[80] Matthews BW (1975). Comparison of the predicted and observed secondary structure of T4 phage lysozyme. Biochim Biophys Acta BBA Protein Struct.

